# Convolution Neural Networks and Self-Attention Learners for Alzheimer Dementia Diagnosis from Brain MRI

**DOI:** 10.3390/s23031694

**Published:** 2023-02-03

**Authors:** Pierluigi Carcagnì, Marco Leo, Marco Del Coco, Cosimo Distante, Andrea De Salve

**Affiliations:** Institute of Applied Sciences and Intelligent Systems 1, National Research Council of Italy, 80078 Pozzuoli, Italy

**Keywords:** assistive technology, MRI, medical image analysis, computer-aided diagnosis, masked auto-encoders, deep learning, vision transformers

## Abstract

Alzheimer’s disease (AD) is the most common form of dementia. Computer-aided diagnosis (CAD) can help in the early detection of associated cognitive impairment. The aim of this work is to improve the automatic detection of dementia in MRI brain data. For this purpose, we used an established pipeline that includes the registration, slicing, and classification steps. The contribution of this research was to investigate for the first time, to our knowledge, three current and promising deep convolutional models (ResNet, DenseNet, and EfficientNet) and two transformer-based architectures (MAE and DeiT) for mapping input images to clinical diagnosis. To allow a fair comparison, the experiments were performed on two publicly available datasets (ADNI and OASIS) using multiple benchmarks obtained by changing the number of slices per subject extracted from the available 3D voxels. The experiments showed that very deep ResNet and DenseNet models performed better than the shallow ResNet and VGG versions tested in the literature. It was also found that transformer architectures, and DeiT in particular, produced the best classification results and were more robust to the noise added by increasing the number of slices. A significant improvement in accuracy (up to 7%) was achieved compared to the leading state-of-the-art approaches, paving the way for the use of CAD approaches in real-world applications.

## 1. Introduction

Dementia is a general term used to describe a premature deterioration of cognitive function beyond biological aging. Alzheimer’s dementia (AD) is the most common form of dementia (70% of cases). It alters memory, thinking, and behavior and gradually affects daily activities and functions. It is an irreversible and complex neurological disorder for which there is no generally effective medical treatment. However, early detection of associated cognitive impairment allows the provision of preventive medications to slow down the progression of the disease [1]. The traditional method for diagnosing Alzheimer’s dementia is to observe people with mild cognitive impairment (MCI) and assess cognitive changes over the years. In this way, doctors can diagnose AD only when the symptoms are evident, and the disease has already reached an advanced stage. On the other hand, AD results from the progressive loss (degeneration) of brain cells. This degeneration can show up in brain scans when symptoms are very mild or even before they occur [2].

Structural imaging techniques such as magnetic resonance imaging (MRI) visualize the structures of the brain and can reveal the loss of neurons and their connections (atrophy) as well as damage in specific regions (e.g., hippocampus) [3]. MRI has made it possible to obtain a three-dimensional (3D) reconstruction of brain structures and to measure the size of the hippocampus and related regions. As a result, MRI-based diagnostics have become an integral part of clinical practice in the diagnosis and evaluation of dementia [4,5]. However, this is a difficult and subjective task that requires a high level of expertise to correctly analyze the images, as neuropathologists examine large brain areas to identify distinct and finely differentiated morphologies [6]. It is also tedious and time-consuming, can lead to differing opinions among experts, and has a slow analysis throughput, making MRI impractical for routine examinations [7]. Computer-aided diagnosis (CAD) can help overcome these drawbacks. Sometimes multimodal data (MRI, positron emission tomography PET and genetic analysis) [8] are used, but this makes the process more complex because several types of regularizations have to be introduced. For this reason, CAD approaches based on a single data modality, and MRI in particular, are the most promising [9].

Existing MRI-based CAD approaches can use the entire 3D brain volume [10] or a series of 2D slices extracted from it [11]. The initial studies relied on traditional algorithmic pipelines (hand-crafted features combined with shallow classifiers) [12]. More recently, following the trend in medical imaging [13,14,15], Deep Learning (DL) is the most common method for automatic brain feature extraction. Since it depends on many training parameters, using DL on 3D brain volumes significantly increases the computational cost [10]. In addition, the availability of 3D data is limited, and its use may suffer from the curse of dimensionality [16], limiting the ability to create accurate models. In addition, pre-trained 3D models are not as widely available as 2D models (trained on huge image datasets) [17]. For the above reasons, deep models based on 2D data have the potential to achieve better accuracy in this domain, but unfortunately, there are still several related open issues:

1. Most existing approaches for binary classification (dementia vs. normal) were tested on 2D MRI slices randomly sampled from the available 3D data without considering to which subject they belonged (slice-level data split strategy) [18,19]. This means that slices belonging to the same subject can occur in both the training and testing processes; in this way, the test data can have a distribution more similar to that of the training set than would be expected from new data belonging to new subjects. This is the well-known data leakage problem in machine learning [20] that has called into question the validity of many previous MRI-based CAD studies and made their use in actual clinical screenings uncertain [21]. The few studies that perform classification of neurologic diseases using MRI and with no data leakage are listed and discussed in [11,22] where it emerges that automatic classification ability is still unsatisfactory to make MRI-based CAD useful in clinical practice.
2. How many 2D slices should be extracted from the available 3D MRI volumes is an open question. Increasing the number of slices per subject may add a little discriminatory information hidden in a larger amount of useless data. The only benchmarks found in the literature are those where the number of slices per subject was fixed a priori (usually 8). The ability of the classifiers to handle this has therefore not been studied at all.
3. Deep-learning models and, in particular, convolutional neural networks (CNN) have revolutionized computer vision, but the most powerful recent CNN models have not yet been explored for AD diagnosis from MRI data. This may be due to the complexity of models’ implementation, data preparation, and validation techniques used in the machine learning community [23].
4. Recent findings in machine learning beyond Convolutional Neural Networks have also not been tested. It has been shown that the mechanism of self-attention can be a viable alternative for building image recognition models [24]. It can be used to direct attention to key areas in the image to obtain high-level information, but as far as we know, this research direction has been less explored in CAD. Recently, there have been some groundbreaking experiments with 3D brain data, but there is no work addressing AD-related issues [25].

In this work, an attempt is made to overcome the above drawbacks with the aim of improving the automatic detection of dementia in MRI brain data. The hypothesis is that the 2D slices extracted from MRI brain data contain relevant information for dementia detection, but unfortunately it is embedded in a large amount of structural data and cannot be fully highlighted by the state-of-the-art classification approaches. Therefore, the key idea is to apply more powerful approaches that can better characterize the data distribution, retain useful information for dementia detection and discard useless ones. For this purpose, the proven pipeline combining the registration, slicing and classification steps has been used. The contribution of this research is that, for the first time, we have explored three of the latest and most promising CNN architectures and two Vision Transformers (ViT) [26] based approaches for mapping input images to clinical diagnosis. In particular, the Resnet [27], DenseNet [28], and EfficientNet [29] architectures were tested. They are currently among the best performing in image classification tasks and have been proven in many other medical image analysis applications [30]. On the other hand, two transformer-based architectures have been implemented: self-attention learners called Masked AutoEncoders (MAE) [31], which are able to automatically highlight relevant regions in brain images, and data-efficient image transformers (DeiT) [32,33], which use a renewed training procedure and require far fewer data and computational resources to build a powerful image classification model. Transformer-based architectures have recently achieved remarkable success and have shown excellent performance on a wide range of image-processing tasks. Transformer-based architectures rely entirely on self-attention mechanisms to establish global and local dependencies between inputs and outputs [34].

Experiments were performed on two large, publicly available datasets and showed a significant improvement in subject-level classification compared to the leading approaches in the state of the art. Furthermore, for each CNN and ViT approach, an evaluation was performed on several benchmarks to assess knowledge extraction and generalization capabilities when the number of slices per subject changes (4, 8, and 16 slices were considered). To allow a fair comparison of classification methods (which is the goal of this paper), 3D volume registration and 2D slice extraction were performed using the same approaches as comparative works in the literature. Noise-related reliability introduced by MRI acquisition with various scanning devices is beyond the scope of this paper. In this regard, the reader may refer to several papers addressing the role of entropy in brain MRI data [35,36], and the effectiveness of 3D data registration [37]. The remainder of the paper is organized as follows: Section 2 describes materials and methods, while Section 3 reports experimental results. Finally, Section 4 concludes the paper.

## 2. Materials and Methods

Two publicly available datasets will be used in the experimental phase, namely the Alzheimer’s Disease Neuroimaging Initiative (ADNI) database (adni.loni.usc.edu, accessed on 31 January 2022) and the Open Access Series of Imaging Studies (OASIS) database (www.oasis-brains.org, accessed on 31 January 2022). The ADNI initiative, involving several centers, was launched in 2004 with the goal of developing biomarkers for the early detection and tracking of AD. Over time, several types of longitudinal data have been collected: demographic data, magnetic resonance imaging (MRI), positron emission tomography (PET) images, genomic sequence variations, and clinical and cognitive assessments. The dataset consists of 4 subsets and in this work, T1-weighted MRI data from subset 2 (namely ADNI-2) were used. ADNI-2 includes longitudinal follow-up of 391 subjects from the 2 previous ADNI phases and recruitment of 780 new participants, resulting in a total of 1171 subjects [38]. The OASIS dataset [39] includes cross-sectional (OASIS -1) and longitudinal (OASIS -2) T1-weighted MRI, longitudinal/multimodal (OASIS -3), and clinical (OASIS -4) data. This work used the cross-sectional collection of 416 subjects aged 18 to 96 years provided by OASIS -1. For each subject, 3 or 4 individual scans were obtained in a single session. From both datasets, 200 subjects were used: 100 patients clinically diagnosed with very mild to moderate Alzheimer’s disease (AD) and 100 healthy controls (or normal controls - NC). They are the same as those used in [22]. Table 1 contains demographic details for both datasets used in the experimental sessions.

**Figure 1:**
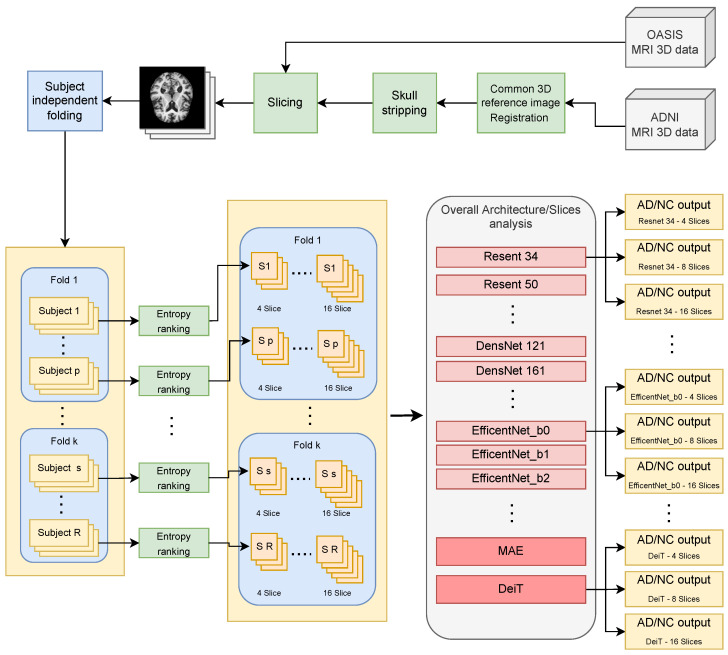
Research workflow: the input 3D data are preprocessed and divided into slices. Then, a k-folding is performed, taking care not to insert slices from the same subject into more than a single fold. The slices are ordered sequentially by their entropy value and prepared in groups of 4, 8, and 16 slices. Finally, the tests are performed in a k-fold manner through the different architectures working on different subsets defined by the number of slices retained per subject.

To make the evaluation as fair as possible, all tests followed the strict workflow shown in Figure 1 and explained in more detail below.

The first step was data preparation. For the ADNI-2 dataset, starting from a T1-weighted 3D image, a processing step was performed to co-register the input to the “Montreal Neurological Institute standard template space”, commonly referred to as MNI152 (at a voxel size of 1 mm), available in the FSL [40] package version 6.0.3, using the SyN algorithm included in the ANTs [41] package (version 2.1.0) with default parameters. Brain tissue in the MRI-registered image was then isolated from non-brain tissue using the brain mask of the standard template space. For the dataset OASIS -1, available registered data from standard post-processing procedures (e.g., atlas registration, bias field correction) were used directly to allow the fairest possible comparison with existing methods. Based on the registered volumes, 2D slices were extracted for both datasets (slicing).

The slices were then split into folds for cross-validation, preserving the information about the subject to which they belong. In this way, slices belonging to one subject can appear only in the training or validation sets (i.e., no data leakage is introduced). Then, slice selection was performed based on the amount of information carried on, i.e., by calculating the Shannon entropy $E_{S}$ for each axial slice. In particular, the following formula was used:

(1) $$E_{S}=\sum_{k}p_{k}\log_{2}\left(p_{k}\right)$$

where *k* is the number of grey levels in the slice and $p_{k}$ is the probability of occurrence for grey level k estimated as the relative frequency in the image. Then, for each subject, the slices were ordered in descending order based on their entropy values, and finally, the *M* axial slices (varying depending on the experiment to be performed) that had the highest entropy were selected according to [42,43].

Selected slices are then provided as input to the end-to-end classification pipeline using DL strategies. Various CNN architectures and the recently introduced MAE and DeiT have been tested.

Following the leading literature, k-fold cross-validation (k=5) was performed for all experiments reported in this section. The most difficult problem of classifying slices according to a subject-level split was addressed. Each model was tested on the ADNI-2 and OASIS -1 datasets on benchmarks created using a different number of slices per subject (M∈{4,8,16}) extracted by exploiting the Shannon entropy value.

It is worth noting that the number of slices is not a hyperparameter of the models. To understand how each model handles the information embedded in noisy data, incremental sizes of benchmarks were established. For this purpose, the series of powers of 2 was chosen, taking into account that most previous works used 8 slices per subject.

For both the CNN and ViT architectures, fine-tuning was performed using models trained on the Imagenet dataset. Data augmentation strategies were used to avoid overfitting during training. In addition, each input slice image was adjusted to a joint resolution of 224×224 pixels using bicubic interpolation. After several experimental tests, different transformations were applied to the input images for all considered CNN architectures, more precisely: random rotation of $5^{\circ }$; random horizontal shift with an image width fraction of 0.05; random vertical shift with an image height fraction of 0.10; random scaling with a scaling factor in the range (1.05, 1.1). Finally, the SGD optimizer was used with an initial learning_rate = 0.001, a momentum of 0.9, and a decay of the learning rate by a factor of 0.1 every 30 epochs. The maximum number of training epochs was set to 100. The ViT architectures used the training hyperparameters and image augmentation strategies based on the *cutmix* and *mix up* approaches as described in the original paper implementation of [31,32].

A brief introduction to the deep models used is given below.

### 2.1. Convolutional Neural Networks

Convolutional neural networks (CNN) have become the standard for most computer vision tasks over the past decade.

CNNs gradually add a series of convolutional layers to a shallow neural network, extracting high-level features from the input image and passing them to the fully connected layers responsible for low-level analysis and final decision. The ability of the convolutional layers to retrieve effective features that can well characterize the image under study guarantees a non-negligible advantage compared to the classical methods based on hand-crafted features. In fact, this approach has allowed an improvement in the generalization capabilities and consequently in the accuracy of the results, which cannot be achieved by classical methods. On the other hand, these amazing capabilities have their price in terms of memory and computational requirements related to the large number of parameters that need to be trained. Today, the scientific community is mainly focused on building large datasets and introducing new models capable of performing well on increasingly challenging tasks related to real-world problems [44]. Among the models that have given a boost to the field of Deep Learning worldwide, some have already been tested in medical imaging [45], but few have been tested for brain MRI analysis [46]. In the following, we detail the models presented and tested for the first time in this work for detecting AD in MRI slices.

#### 2.1.1. ResNet

Convolutional networks used by the computer vision community are getting deeper and deeper every year. Unfortunately, when the depth of the network exceeds certain limits, the accuracy goes into saturation and then rapidly decreases due to the vanishing gradient. This makes it impossible to train very deep networks and use them for complex problems. To overcome this degradation problem, ResNet architectures [27] introduce the *residual learning framework*, which exploits shortcut identity connections between convolutional layers (*Conv Block*) to reinvigorate information flow. This has been shown to effectively mitigate degradation phenomena, allowing the use of very deep networks and providing a non-negligible gain in accuracy. Residual units are typically non-linear, which prevents a ResNet from expanding exponentially into separate subnetworks. In this work, four residual network architectures were tested: ResNet34, ResNet50, ResNet101, and ResNet152, where the number indicates the number of layers that can actually be trained.

#### 2.1.2. DenseNet

Backpropagation algorithms and gradient-based methods used for training deep neural networks use the computation of the partial derivatives of the loss function with respect to the weights of the network to provide updates to the learnable parameters of the network. As the depth of the network increases, the value of the gradient decreases exponentially, leading to the vanishing gradient problem. DenseNet [28] attempts to address this problem by ensuring maximum information (and gradient) flow by connecting each layer directly to each of the following layers. In other words, rather than entrusting the network’s representational capability to extremely deep or wide architectures, DenseNet leverages feature reuse. Unlike ResNet, which uses summation to combine features before they reach the layers, DenseNet uses the concatenation of feature maps. However, for this process to be feasible, the feature maps must not change the size, which is the case with CNN downsampling layers. To achieve this, the DenseNet is divided into dense blocks within which the feature maps have a constant size. Variation in the dimensions of the feature maps is achieved by transition layers, each consisting of a convolutional layer and a pooling layer, between two adjacent dense blocks.

This approach has several advantages: First, the proposed connection strategy requires fewer parameters than a corresponding traditional CNN. Moreover, DenseNet involves narrower layers (e.g., 12 filters) than other CNN approaches and the addition of a small number of new feature maps. Finally, the training phase also benefits from this structure since each layer can directly access the gradients. In this work, four DenseNet network architectures were tested: DenseNet121, DenseNet161, DenseNet169, and DenseNet201, where the number indicates the convolutional layers in four DenseBlocks and transition layers (to which the input and the last fully connected layer must be added to reach the number indicated in the model name).

#### 2.1.3. EfficientNet

Scaling up a network is not a trivial task. Both depth-wise and width-wise approaches can be used to increase the network size and hopefully make it more powerful. Using higher-resolution inputs is also a viable way to further improve the results. Unfortunately, all of these solutions lead to a rapid increase in the parameters used and consequently in the computational and memory resource requirements. The authors of [29] propose a new scaling method that aims to scale a given network along all its dimensions (i.e., depth/width/resolution) using a single *compound coefficient*. The paper proposes a baseline network to be scaled up to obtain a family of networks, called EfficentNets, capable of achieving higher accuracy compared to other state-of-the-art solutions. Based on some previous research that showed a certain relationship between network width and depth, the authors developed the compound scaling method over a simple idea: higher-resolution images need deeper networks to increase the receptive field and additional channels to capture the fine-grained patterns present in the image. Such compound scaling is based on three constant coefficients, α,β,γ, which are related to the architectural choices of the network in terms of depth, width and size of the input image, respectively. The main component of EfficientNets is a residual block with an inverted structure compared to the residual blocks used in ResNet (i.e., a narrower number of channels are used in the information flow for efficiency reasons), to which squeeze-and-excitation optimization is also added. In this paper, eight different architectures were tested, scaled from the baseline version, named EfficientNet_b0, with different compound coefficients.

### 2.2. Visual Transformers

In recent years, the field of Natural Language Processing (NLP) has seen significant progress with the introduction of transformers [47]. Such an approach is characterized by high generality and computational efficiency, which led to the idea of Visual Transformers (ViT) [48] in the field of image processing. ViT keeps the generic architecture unchanged, making only the minor changes necessary to process images. Briefly, the input image is divided into a set of *visual tokens* embedded in a set of encoded vectors including their position in the image. The vectors are sequentially fed into the *transformer encoding network*, which consists of three key elements: *Layer Norm*, *Multi-head Attention Network* (MSP), and *Multi-Layer Perceptrons* (MLP). The MSP is dedicated to the generation of the attention maps from the provided visual token, the layer norm makes the model adaptable to the variations among images and, finally, MLP is a two-layer classification network. These steps can be repeated multiple times into the *transformer encoding network* until the final MLP block, known as *MLP head*, which is the output of the transformer and usually feeds a *softmax* function to enable the classification task. As stated before, visual transformers keep a higher generality and have a non-negligible advantage since the primary embedding is context agnostic. The price is a drawback of the larger amount of training data required to get the same performance as CNN. Moreover, it has been proved that they are able to attend to image areas that are semantically relevant for classification. Beyond ViT base implementation, some improvements have been recently proposed and two of the most promising approaches are the Masked Auto-Encoders (MAE) [31] and Data-efficient image Transformers (DeiT) [32].

#### 2.2.1. MAE

Masked Auto-encoders (MAE) [31] are self-supervised learning approaches based on an asymmetric encoder-decoder architecture. They take advantage of two main ideas: masking a given percentage of the image patches and keeping this percentage high. This approach employed the ViT [48] architecture for the encoding side (ViT are highly suitable for the masked patch paradigm), enabling the training of large models efficiently and effectively. The masking paradigm starts from the heavy spatial redundancy of image where the recovery of a patch can be achieved by its neighbors even with a little knowledge at a high level of the whole scene. On the other hand, masking a substantial portion of the image forces the model to face a more challenging self-supervised problem, leading to a holistic understanding of the image. Going into details, the solution proposed in [31] is based on an asymmetric encoder-decoder design where the encoder takes in input a subset of the image patches ignoring the masked ones. On the other side, a lightweight decoder reconstructs the input from the provided latent representation. The use of just the non-masked portion of the image patches, together with the use of a lightweight decoder, keeps the computational requirements low, boosting the training phase: an aspect that is particularly profitable for data-hungry models like ViT. In this paper, we performed transfer learning for the classification downstream task using the ViT-Base pre-trained checkpoint available at https://github.com/facebookresearch/mae, accessed on 31 January 2022. The Vit-Base was proposed in [48] and it is characterized by an embedding dimension D=768, number of heads h=12 and number of layers L=12 for a total 86M learnable parameters.

#### 2.2.2. DeiT

In both above mentioned ViT and MAE approaches, the promising results in terms of generality, accuracy and computational requirements have to pay the nontrivial drawback related to the required huge amount of data that is not ever available. Data-efficient image Transformers (DeiT) [32] leverage on a training phase based on a teacher-students strategy. More precisely, it makes use of a distillation token ensuring that the student learns from the teacher by means of the attention mechanism of transformers using a convolutional network as a teacher. Knowledge distillation is considered both in form of soft and hard distillation where the latter clearly outperforms the first one in all the experiments. It is also worth noting as the use of a convolutional network as a teacher allows the trained transformers to inherit the inductive bias if compared with transformers trained from scratch.

In this paper, the model pre-trained on the Imagenet dataset and available at (https://github.com/facebookresearch/deit, accessed on 31 January 2022) has been fine-tuned. The architecture design is the one proposed in [48] with no convolutions. The only differences are the training strategies and the distillation token. For the pre-training, only a linear classifier was used.

In the study presented in this paper, the best results were obtained, among all experiments, using the DeiT-B model with 224×224 input image size. This model follows the same architecture as ViT-Base but with a different training strategy that does not use an MLP head for the pre-training but only a linear classifier.

## 3. Results

In this section, the experimental results on the two datasets described in Section 2 are reported. The evaluation was carried out in terms of accuracy given that the datasets used are balanced. It has been computed as follows:

(2) $$Accuracy=\frac{\left[TP+TN\right]}{\left[TP+TN+FP+FN\right]}$$

where TP stands for True Positives (slices belonging to an AD subject correctly classified as AD) and TN stands for True Negatives (slices belonging to a control subject correctly classified as non-AD).

All experiments have been performed in Pytorch [49] deep learning framework using an NVIDIA RTX 3090Ti GPU card equipped with 24GB of RAM.

### 3.1. Results by CNN

Table 2 reports the classification results obtained using the CNN models.

The best classification results are highlighted in bold in each column. Going deeper, the DenseNet201 model generated the best results for the ADNI-2 dataset in the 4 slices per subject case (accuracy 69.751%), while DenseNet161 and EfficientNet_b0 scored best in the 8 slices (accuracy 70.190%) and 16 slices (accuracy 69.534%) per subject cases, respectively. On the OASIS-1 dataset, the ResNet-152 model performed best with 8 and 16 slices, with an accuracy of 71.124% and 69%, respectively. The DenseNet169 was best in the case of 4 slices extracted per subject with an accuracy of 73.501%.

**Figure 2:**
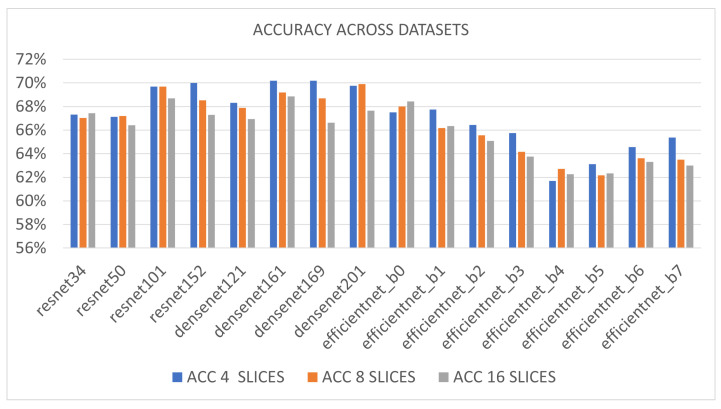
Accuracy across datasets of tested CNN models.

In Figure 2, the mean accuracy across datasets is reported. It represents the accuracy reported by each model among validation folds on both datasets.

The ResNet152, DenseNet161 and DenseNet169 models gathered the best scores (a few more than 70%) when 4 slices per subject were extracted (blue vertical bars). In the case of 8 slices per subject (orange vertical bars), the model that experienced the best accuracy clearly was the DenseNet201 model (mean accuracy almost 70%) whereas in the case of 16 slices per subject (grey vertical bars), the most accurate model was the DenseNet161 (mean accuracy more than 69%). Overall, all the DenseNet models provided satisfying outcomes in all three experimental cases (average accuracy across experiments and models 68.68% against EfficientNetand ResNet models reaching 64.69% and 68.03%). Satisfactory results were also reached by using deeper ResNet models and the tightest EfficientNet one. From accuracy results in Table 2, it is possible to understand that, for the considered classification problem, CNN requires representing the information on several levels of extracted features. This can be better achieved by DenseNet architectures, which make use of identity connections on each layer. On the other side, ResNet architectures, which have identity connections limited to blocks (of consecutive levels) work very well too, but they are not able to completely get a representation of the information embedded in the data on different datasets and benchmarks. Finally, it emerged that none of the eight EfficientNet architectures provided excellent classification outcomes. The main reason for this drawback could be the impossibility to transfer the information flow along layers through inverted residual blocks relying on a narrowed number of channels. It might be possible to conclude that this kind of architecture is not suitable for modeling such a complex problem as the classification of brain MRI images.

### 3.2. Results by Visual Transformers

This section reports the results obtained by using two of the most recent and promising visual transformer architectures that were never tested for the scope of AD diagnosis from MRI images. Table 3 resumes the accuracy results for MAE and DeiT architectures. Both architectures were tested on both datasets in the 4, 8 and 16 slices per subject cases. DeiT outperformed MAE in each performed experiment with an accuracy of 77% for the 4 slices experiment, 75.937% for the 8 slices experiment and 75.625% for the 16 slices experiments, respectively. It is worth noting that, differently from CNN architectures, the accuracy of visual transformers had fewer variations than CNN when the number of slices increased. This might be due to the ability of the embedded self-attention mechanism to discard useless information introduced by adding more slices; they were anyhow able to extract robust knowledge from the available data although the classification problem became more complex. In general, transformers architectures performed better than CNN ones with a difference up to more than 7% between the most performing architectures in each experimental phase (e.g., 69.751% by DenseNet201 against 77% by DeiT in the case of 4 slices per subject).

Summing up, through the experimental phases, it is possible to understand that the self-attention mechanism of ViT resulted, so far, the winning key for this complex classification problem. In our opinion, this is the main scientific funding of the manuscript. The model splits the images into a series of positional embedding patches, which are processed by the transformer encoder. This allows ViT to relate different positions of the pixel values in the slides without requiring image-specific biases and then making it possible to recognize anomalies due to dementia independently of their spatial locations. This way, self-attention allows a very encouraging increase in classification accuracy that could bring to the exploitation of automatic diagnosis in real clinical practice.

These are very encouraging results. The downside is that visual transformers have a more significant number of learnable parameters with respect to CNN models (DeiT has 86M parameters, DenseNet201 has 20M parameters, ResNet101 has 40M parameters, and ResNet152 has about 60M).

### 3.3. Comparisons to Leading Approaches

This section compares the best results achieved by the deep architectures, reported in previous sections, with the leading state-of-the-art approaches. There are only a few approaches that use the split of data by subject and therefore without a data leakage problem. They include 3 architectures introduced and tested in [22] on both ADNI-2 and OASIS-1 datasets by extracting 8 slices per subject. The architectures are two different variants of VGG and a ResNet-18. The first model, named VGG16-v1 consists of five convolutional blocks followed by three fully connected layers which were fine-tuned.

The second model, VGG16-v2, includes a global average pooling layer after convolutional blocks, and all the layers were fine-tuned. Finally, in [50] a different VGG16 model was introduced for the scope: a global average pooling (GAP) layer was used instead of fully connected (FC) layers and the last classification layer with a ‘softmax’ activation was added.

Tests were performed in the case of 10 slices per subject. All the comparing works used entropy criteria for selecting relevant slices. In Table 4, the proposed approach is compared to the best-performing approaches in the literature (only works in which data leakage has been avoided are considered). It is worth noting that the same k-fold with k = 5 has been used for a fair comparison. Details on the CNN architectures can be found in the respective papers [22,50].

**Figure 3:**
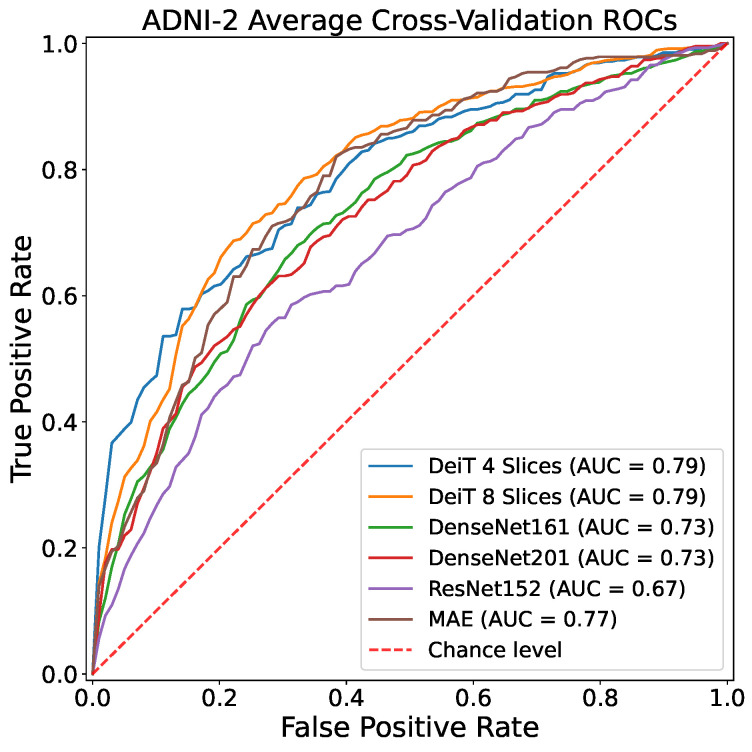
Cross-Validation ROCs related to ADNI-2 dataset.

**Figure 4:**
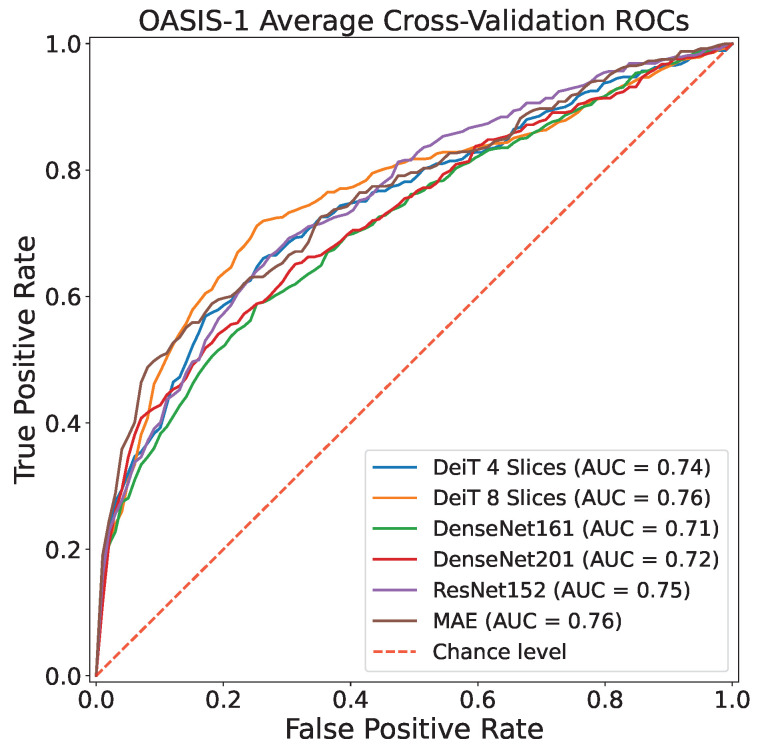
Cross-Validation ROCs related to OASIS-1 dataset.

Table 4 clearly indicates the improvement introduced by the CNN models considered in this work with respect to previous approaches proposed in the literature. Results reveal how the tested DL architectures, and especially the visual transformer architectures (DeiT), are the leading in MRI 2D slices classification in the case of no data leakage, making a big step towards actual exploitation of CAD systems in real-life AD diagnosis. This is corroborated also by observing ROC curves reported in Figure 3 and Figure 4.

## 4. Conclusions

In this work, three different CNN strategies and two visual transformers were tested for the first time to classify 2D MRI brain data as belonging to subjects with Alzheimer’s dementia or healthy. The proposed approach started from 3D MRI volumes and extracted the 2D slices with the highest entropy score after 3D registration and skull stripping operations. Subsequently, subject-level partitioning was performed to avoid the common drawbacks of data leakage. CNN and ViT architectures were then trained and tested on two publicly available datasets and three different experimental cases, i.e., considering 4, 8, and 16 slices per subject. The results showed a significant improvement in accuracy compared to the state-of-the-art and paved the way for the use of CAD approaches in real applications.

There are some limitations to the study. Only a few of the newer deep-learning models were tested. They were used as presented in the literature for traditional image classification tasks. No task-specific changes to the models nor hyperparameter optimization were carried out. In addition, there is a persistent discrepancy between the accuracy of the training (more than 90% in all experiments) and test data. Finally, there is a lack of evaluation of the positions of the extracted slices from brain volumes. The entropy criteria can pull out very close and redundant slices indeed and furthermore, the informative content for the AD classification may not necessarily be related to the data variability.

Future work will look at evaluating different criteria for extracting layers (beyond entropy) and at using the proposed pipeline to account for informative content of each slice to further increase the accuracy of diagnosis, but also to reduce the data content of the inputs, making CAD more suitable for use in real-world applications. The use of tiny visual transformers will be also addressed to obtain good classification based on fewer parameters. Tiny versions have parameters comparable to mid-sized CNNs such as the ResNet50 (less than 20 million parameters), and their accuracy should be carefully evaluated in this challenging application domain. The use of visual transformers on multimodal brain data (not just MRI) could also be an interesting research direction to advance this field. Visual transformers could automatically account for relationships that occur within and between modalities to further increase diagnostic accuracy. Finally, the proposed pipeline could be also used in other medical fields (e.g., cancer detection and grading in histopathology images).

## Figures and Tables

**Table 1:** Details of the datasets.

Dataset	Classes	Subjects	Gender	Age (in Years)
			(Women/Men)	(Range; Mean ± SD)
ADNI-2	AD	100	44/56	56–89; 74.28±7.96
	NC	100	52/48	58–95; 75.04±7.11
OASIS-1	AD	100	44/56	62–96; 76.70±7.10
	NC	100	52/48	59–94; 75.50±9.10

**Table 2:** AD/NC classification accuracy gathered by CNN. The bold style highlights the best classification results in each column.

Method	Cross-Validation Accuracy (%)					
	ADNI-2			OASIS-1		
	4 Slices	8 Slices	16 Slices	4 Slices	8 Slices	16 Slices
ResNet34	65.500	66.874	67.470	68.126	67.190	67.409
ResNet50	65.752	66.374	65.624	68.502	68.000	68.653
ResNet101	68.751	69.500	69.531	70.626	69.875	67.842
ResNet152	67.876	65.937	65.594	72.127	**71.124**	**69.000**
DenseNet121	66.378	65.438	66.219	70.252	70.312	67.654
DenseNet161	69.375	**70.190**	69.253	71.001	68.190	68.470
DenseNet169	66.873	66.564	65.468	**73.501**	70.814	67.780
DenseNet201	**69.751**	69.626	68.626	69.754	70.190	66.690
EfficientNet_b0	69.002	69.502	**69.534**	66.002	66.502	67.341
EfficientNet_b1	66.001	66.066	66.218	69.502	66.314	66.470
EfficientNet_b2	66.124	65.687	65.594	66.752	65.435	64.562
EfficientNet_b3	64.626	63.626	63.408	66.878	64.690	64.126
EfficientNet_b4	61.376	60.502	61.905	62.002	66.938	62.624
EfficientNet_b5	60.249	58.500	59.624	66.004	65.814	65.034
EfficientNet_b6	65.877	63.626	63.970	63.252	63.624	62.656
EfficientNet_b7	63.243	61.874	62.534	68.004	65.124	63.470

**Table 3:** AD/NC classification accuracy gathered by Visual Transformers. The bold style highlights the best classification results in each column.

Method	Cross-Validation Accuracy (%)					
	ADNI-2			OASIS-1		
	4 Slices	8 Slices	16 Slices	4 Slices	8 Slices	16 Slices
MAE	73.555	73.125	71.875	72.375	71.875	70.937
DeiT	**77.000**	75.937	75.625	**74.375**	74.562	72.562

**Table 4:** Comparison of the proposed approach with leading ones (with no data leakage issue) in the literature. The bold style highlights the best classification results in each column.

	Method	Cross-Validation Accuracy (%)	
		ADNI-2	OASIS-1
Previous	VGG16-v1 (8 slices) [22]	70.1	66
	VGG16-v2 (8 slices) [22]	66.4	66.1
	ResNet-18 (8 slices) [22]	68.6	68.8
	VGG16 (10 slices) [50]		71.6
Proposed	ResNet152 (4 slices)	67.2	72
	DenseNet201 (4 slices)	69.8	69.8
	DenseNet161 (8 slices)	70.2	68.2
	MAE (4 slices)	73.6	72.4
	DeiT (4 slices)	**77**	74.4
	DeiT (8 slices)	76	**75.6**

## Data Availability

MRI data used in the experiments were downloaded from the public datasets available at https://adni.loni.usc.edu/ (accessed on 31 January 2022) and https://www.oasis-brains.org/ (accessed on 31 January 2022).

## Abbreviations

The following abbreviations are used in this manuscript:

AD	Alzheimer’ Dementia
DL	Deep Learning
NC	normal controls
MRI	Magnetic resonance imaging
ADNI	Alzheimer’s Disease Neuroimaging Initiative
OASIS	Open Access Series of Imaging Studies
CAD	Computer-aided diagnosis
MAE	Masked AutoEncoders
MCI	Mild Cognitive Impairment
MRI	Magnetic resonance imaging
CNN	Convolutional Neural Networks
DeiT	Data-efficient image Transformers
ViT	Visual Transformers
